# Genome-wide analysis of alternative splicing differences in hepatic ischemia reperfusion injury

**DOI:** 10.1038/s41598-024-82846-1

**Published:** 2024-12-28

**Authors:** Yongliang Hua, Xinglong Li, Bing Yin, Shounan Lu, Baolin Qian, Yongzhi Zhou, Zhongyu Li, Zhanzhi Meng, Yong Ma

**Affiliations:** 1 Department of Minimally Invasive Hepatic Surgery, Key Laboratory of Hepatosplenic Surgery, the First Affiliated Hospital of Harbin Medical University, Ministry of Education, Harbin, Heilongjiang China; 2 Department of Pediatric Surgery, Key Laboratory of Hepatosplenic Surgery, the Sixth Affiliated Hospital of Harbin Medical University, Ministry of Education, Harbin, Heilongjiang China

**Keywords:** Biomarkers, Diseases

## Abstract

Alternative splicing (AS) contributes to transcript and protein diversity, affecting their structure and function. However, the specific transcriptional regulatory mechanisms underlying AS in the context of hepatic ischemia reperfusion (IR) injury in mice have not been extensively characterized. In this study, we investigated differentially alternatively spliced (DAS) genes and differentially expressed transcripts (DETs) in a mouse model of hepatic IR injury using the high throughput RNA sequencing (RNA-seq) analysis and replicate multivariate analysis of transcript splicing (rMATS) analysis. We further conducted Gene ontology (GO) term enrichment, the Kyoto Encyclopedia of Genes and Genomes (KEGG) database and the protein-protein interaction (PPI) network. A total of 898 DAS genes (*p* ≤ 0.05) were screened out in the hepatic IR group compared to the sham group, while functional enrichment analysis revealed that DETs and DAS genes were significantly associated with the ATP-dependent chromain, splicesome and metabolic pathways. The expression level of the DAS genes: Gabpb2, Smg1, Tnrc6c, Mettl17, Smpd4, Kcnt2, D16Ertd472e, Rab3gap2, Echdc2 and Ssx2ip were verified by RT-PCR and qRT-PCR. Our findings provide a comprehensive genome-wide view of AS events in hepatic IR injury in mice, enhancing our understanding of AS dynamics and the molecular mechanisms governing alternative pre-mRNA splicing.

**Supplementary Information:**

The online version contains supplementary material available at 10.1038/s41598-024-82846-1.

## Introduction

In many clinical scenarios, hepatic ischemia reperfusion (IR) injury emerges as a prevalent and severe complication, significantly contributing to postoperative morbidity and mortality^1,2^. The initial phase of IR process involves a temporary disruption of the blood supply to the liver, leading to oxygen deprivation. This is followed by the restoration of blood flow and subsequent reoxygenation. Several pathophysiological events contribute to the development and progression of IR-induced liver injury, including oxidative stress, inflammatory responses, calcium-iron imbalances, and cellular apoptosis^3,4^

Alternative splicing (AS) is a widespread post-transcriptional regulatory mechanismthat generates multiple RNA transcript isoforms in response to surrounding signals in higher eukaryotes^5,6^. Splicing an unspliced RNA, which has introns removed and exon linking, can result in the generation of multiple mature mRNAs from a single pre-mRNA^7,8^. Exons in the same gene can be expressed in different combinations, so that a single gene can make different proteins at different times and in different environments, increasing the genetic complexity and adaptability of the system under physiological and pathological conditions^9^. The common types of AS events have been classified as alternate promoter (AP), alternate terminator (AT), skipped exon (SE), retained intron (RI), alternate donor sites (AD), acceptor sites (AA) and mutually exclusive exons (MXE)^10^. AS plays a critical role in cell differentiation, aging, self-renewal and various cellular functions^11,12^.

Growing evidence has revealed that abnormal AS events are closely related to the pathologic process of hepatic IR injury and liver cancer^13,14^. Our study focused on analyzing genome-wide hepatic IR injury on a genome-wide scale using RNA-seq data, with a focus on exploring the relationship between AS and hepatic IR injury. Additionally, we discovered important DAS genes and DETs in the hepatic IR group, distinguishing them from the control group. Then we explored the biological functions and the underlying mechanisms based on the GO term enrichment analysis and the KEGG pathway enrichment analysis. In a word, our findings significantly extend the current understanding and comprehension of IR-induced AS events in the livers of mice.

## Results

### DETs characters of hepatic IR injury

**Fig. 1:**
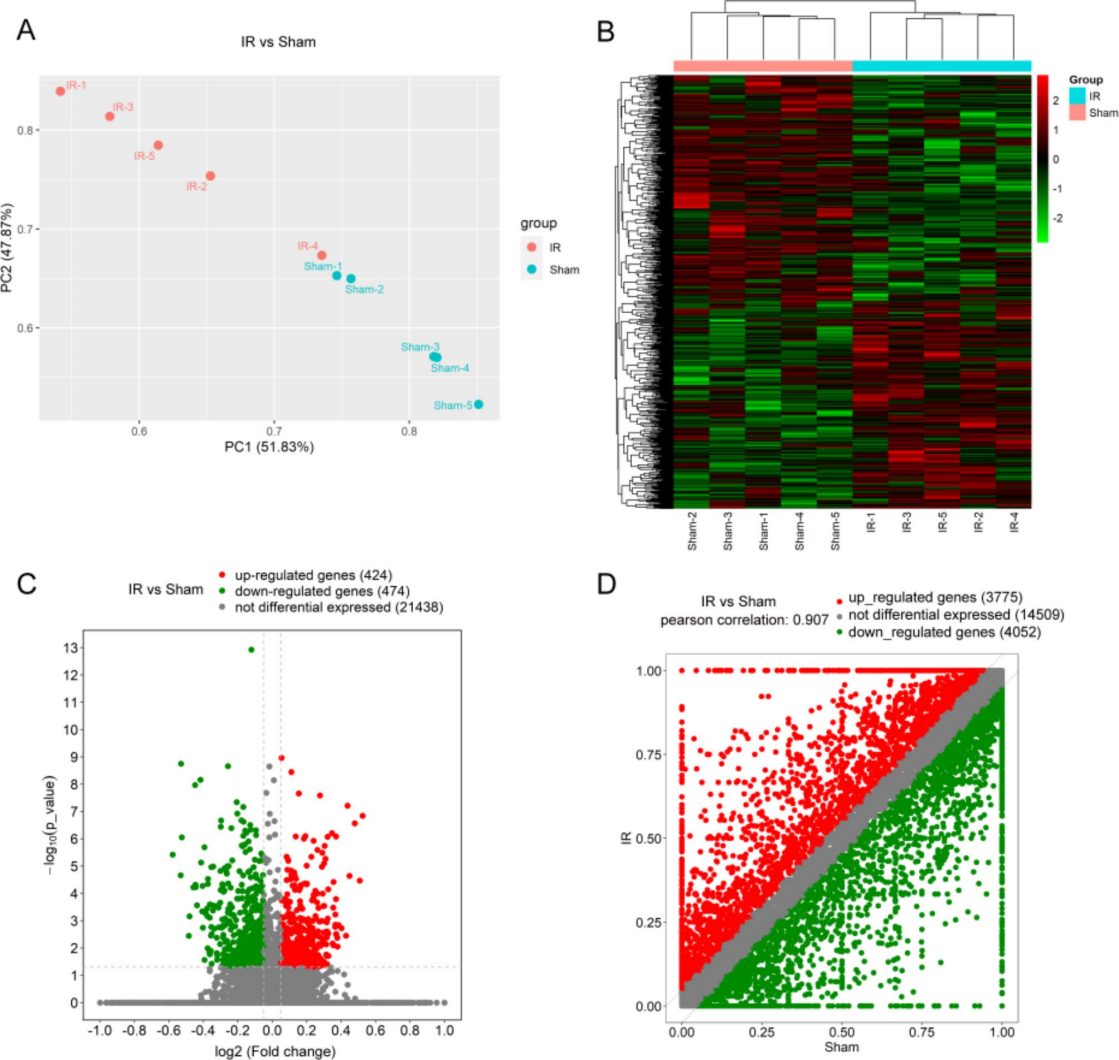
Analysis of RNA-seq data of hepatic IR injury in mice. (**A**) Scatter plot of the PCA analysis for the IR and sham groups. (**B**) Heatmap of hierarchical clustering analysis the ranscript expression patterns in the hepatic IR and sham groups. (**C**) Volcano plots showing differential expression genes in hepatic IR groups compared with sham groups (log2FC ≥ 0.585/log2FC ≤ -0.585 and p ≤ 0.05). (**D**) The scatter diagram showing the levels of mRNA genes with AS events in the hepatic IR and sham groups.

In this study, the RNA-seq method was utilized to assess the expression levels of liver samples and to analyze the transcriptome profiles of differences and AS pattern alterations in the transcripts and mRNAs. The Principal Component Analysis (PCA) revealed a distinct separation between the samples of the two groups, indicating a clear distinction in their underlying characteristics (Fig. 1A).The details of the sequencing data quality of mRNA are shown in Table S1. The reads were uniquely mapped and assembled into transcripts using StringTie software, and the gene transcript expression was quantified using FPKM values (FPKM ≥ 0.5). A total of 203,552,240 reads were generated from five samples in IR group; 209,451,318 reads were generated from five samples in sham groups. Hierarchical clustering was used to demonstrate distinct analysis of transcript expression patterns between the two groups (Fig. 1B). There were 898 DAS genes in the hepatic IR group compared with the sham group, including 424 upregulated genes (log2FC > = 0.585, and *p* ≤ 0.05) and 474 downregulated genes (log2FC <= -0.585, and *p* ≤ 0.05) identified using a volcano plot (Fig. 1C). The cutoff value appears to be ± 0.2. The scatter diagram showed differentially expressed genes with AS events in the hepatic IR group compared to the sham groups (Fig. 1D). Besides the currently known genes annotated in sequence databases, there were far more novel genes/transcripts identified than protein-coding genes in each tissue using RNA-seq, and the name of novel gene/transcript identifier was given by StringTie.

### Overview of AS events analysis

**Fig. 2:**
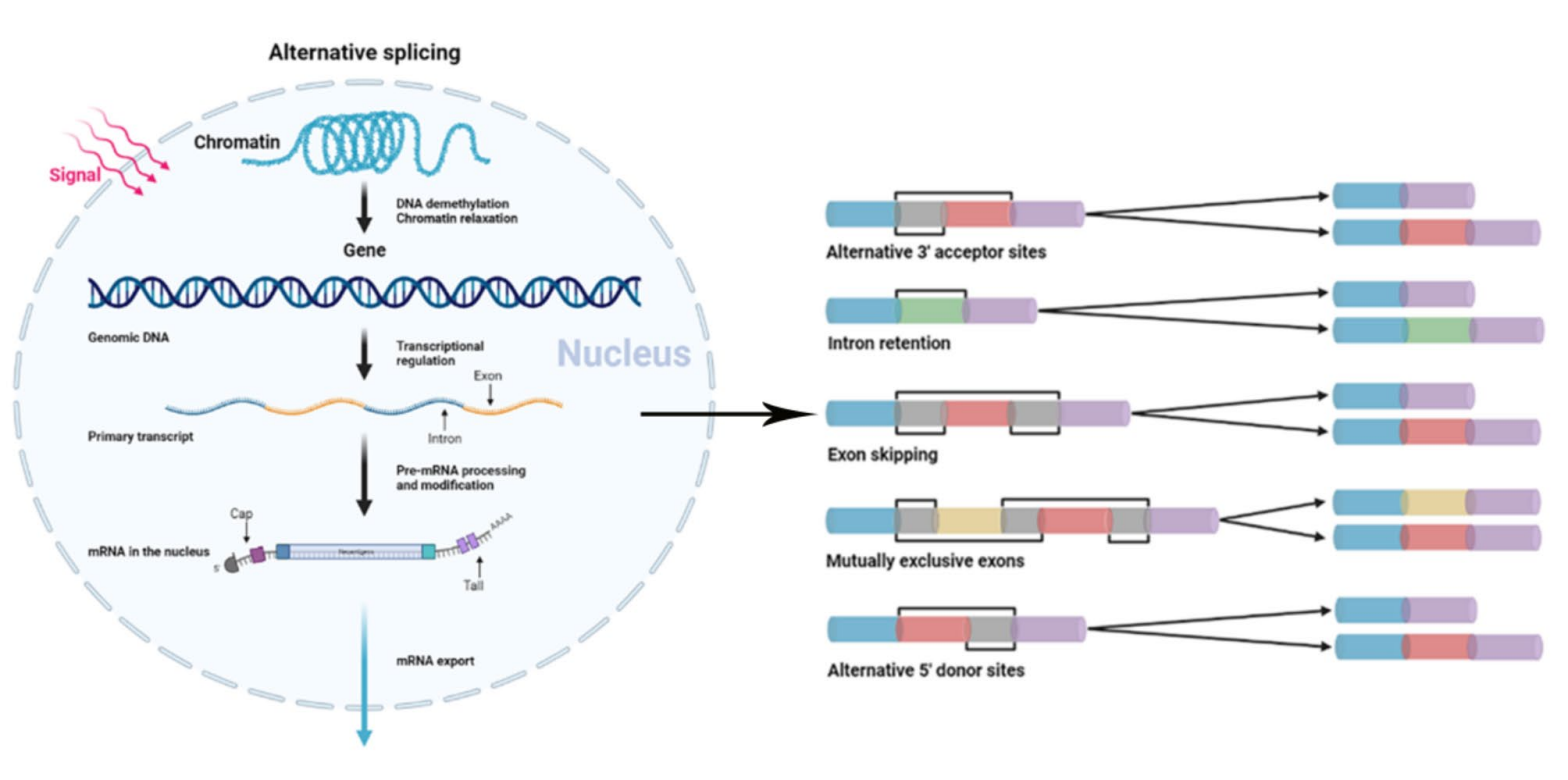
The diagram presents the AS modes in hepatic IR injury.

**Fig. 3:**
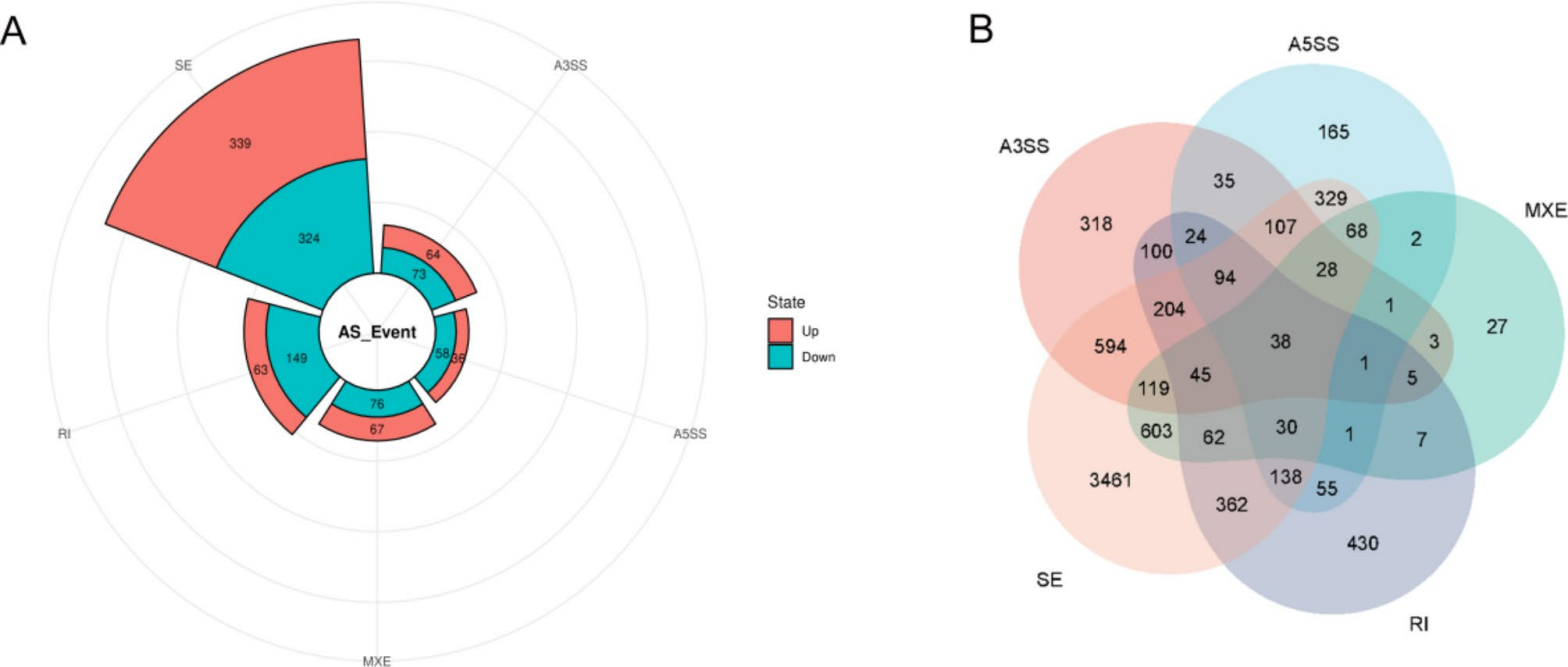
Analysis of DAS genes and distribution of the five main AS events in the hepatic IR and sham groups. (**A**) Number of alternative exons between IR and sham groups in mice (*p* ≤ 0.05). (**B**) The venn diagram shows the distribution of AS events in the hepatic IR and sham groups.

To investigate the differing AS events among the hepatic IR and sham groups, five main types of AS events, including alternative 5’splice sites (A5SS), alternative 3’splice sites (A3SS), skipped exon (SE), retained intron (RI) and mutually exclusive exons (MXE), were analyzed with the program rMATS (Fig. 2A). In comparing the IR group against the sham group, 2229 genes were detected in the A3SS events, 1342 genes were detected in the A5SS events, 1534 genes were detected in the MXE events, 2229 genes were detected in the RI events, and 14,939 genes were detected in the SE events. Furthermore, there were 2171 DET and 1249 DAS genes identified in the IR and sham groups (adjusted p-value < 0.05). According to diff value (IncLevel_IR – IncLevel_Sham), AS events were divided into up-regulated (diff>0) and down-regulated (diff<0) (Fig. 3A). The intersections of the five AS types are presented in Fig. 3A. The numbers of AS events were presented in the Table S2. The intersections of five AS event types were shown in Fig. 3B. The group that included genes from only SE contained 3461 genes and was the most prevalent AS event in comparing IR group versus sham group, whereas MXE was the least prevalent.

### Verification of differential AS genes

**Fig. 4:**
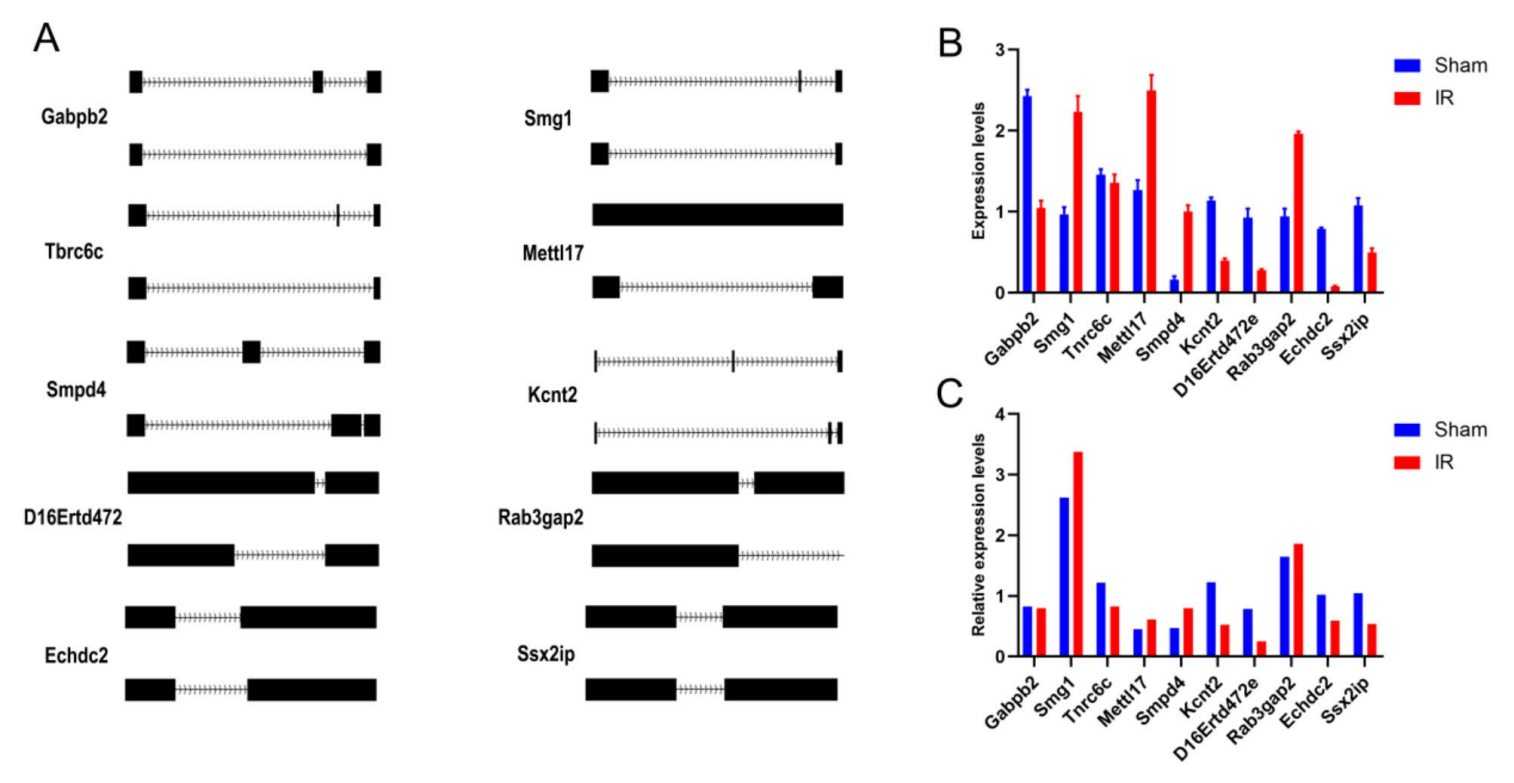
Validation of the expression of DAS genes in the hepatic IR and sham groups. (**A**) RNA sequencing read density plot. (**B**) Validation of AS genes by qRT-PCR. (**C**) RNA-seq of AS genes. Data are shown as the mean ± SD of three independent experiments performed in triplicate.

It was discovered that certain AS events were conserved and had significant differences between IR and sham groups. Some DAS genes were randomly selected from each of five AS events types (*p*<0.05). For example, Gabpb2 (ENSMUSG00000038766.16) and Smg1 (ENSMUSG00000030655.15) were alternatively spliced at the SE patterns and differentially expressed in the IR and sham groups; Tnrc6c (ENSMUSG00000025571.13) and Mettl17 (ENSMUSG00000004561.14) were alternatively spliced at RI patterns in the IR and sham groups; Smpd4 (ENSMUSG00000005899.12) and Kcnt2 (ENSMUSG00000052726.15) were alternatively spliced at different MXE in the IR and sham groups; D16Ertd472e (ENSMUSG00000022864.13) and Rab3gap2 (ENSMUSG00000039318.12) were alternatively spliced at different A5SS in the hepatic IR and sham groups; Additionally, Echdc2 (ENSMUSG00000028601.18) and Ssx2ip (ENSMUSG00000036825.12) different A3SS patterns were found to be differentially expressed between the IR and sham groups (Fig. 4A and Supplemental Fig. 1). These genes were analyzed using RT-PCR and qRT-PCR to validate differential splicing events and expression levels. The results showed that the qRT-PCR was consistent with the results of RNA sequencing (Fig. 4B-C).

### GO and KEGG enrichment analysis

**Fig. 5:**
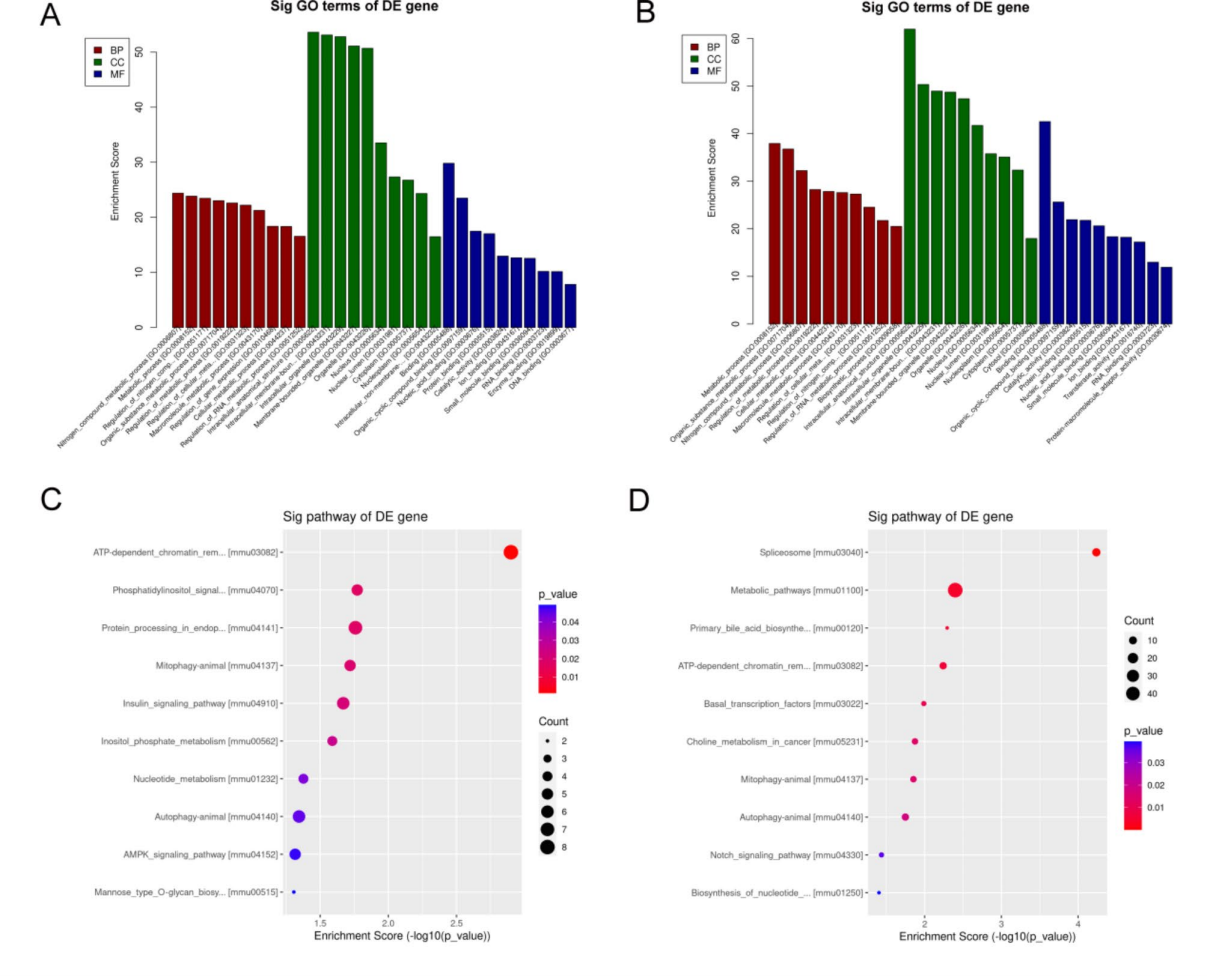
GO and KEGG analyses of DAS genes in the hepatic IR vs. sham groups. (**A**) the top ten significant GO enrichment terms for the up-regulated genes. (**B**) the top ten significant GO enrichment terms for the down-regulated genes. (**C**) the top ten enrichment scores of the enrichment pathway for the up-regulated genes. (**D**) the top ten enrichment scores of the significant enrichment pathway for the down-regulated genes.

To delve deeper into the biological implicationsof differentially expressed alternatively spliced genes between hepatic IR and sham groups, we performed GO and KEGG enrichment analysis. The 30 most enriched GO terms for the three GO categories are presented with a corrected p-value<0.05. Regarding biological processes, for the up-regulated genes, the significantly enriched terms were related to nitrogen compound metabolic process and metabolic process. For the down-regulated genes, the significantly enriched terms were metabolic process and organic substance metabolic process. For the up-regulated and down-regulated genes, regarding cellular components, the significantly enriched terms were intracellular anatomical structure and intracellular organelle. Regarding molecular functions, the significantly enriched terms were binding and organic cyclic compound binding (Fig. 5A-B).

KEGG pathways were obtained based on the enrichment score principle. According to the enrichment score, the top ten pathways with the highest enrichment score (-log10 (p value)) in the compared groups were listed and ranked. For the up-regulated genes, the significantly enriched terms were the ATP-dependent chromatin pathway, phosphatidylinositol signal pathways and protein processing pathways (Fig. 5C). For the down-regulated genes, the significantly enriched terms were the splicesome signaling pathway, metabolic and primary bile acid biosynthetic signaling pathway (Fig. 5D).

### Gene interaction network analyses

**Fig. 6:**
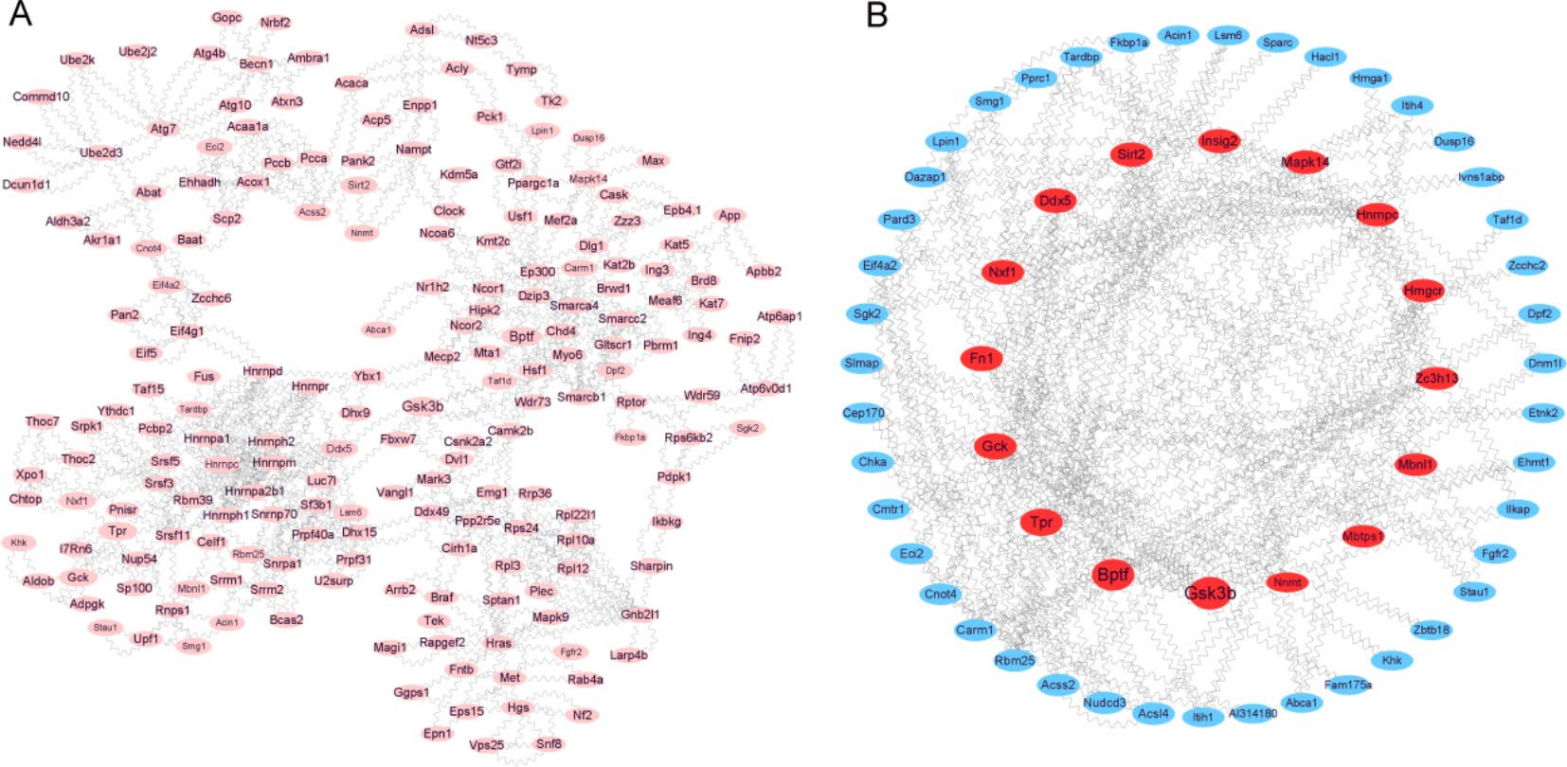
AS events interaction network and the distribution of the DAS genes in the PPI network. (**A**) The distribution of the DAS genes in the PPI network (*p* ≤ 0.05). (**B**) The distribution of differential mRNAs with DAS events (fold change ≥ 1.5, p value ≤ 0.05).

Protein-protein interaction (PPI) networks were constructed for all the differentially expressed alternatively spliced genes associated with hepatic IR injury using the STRING database. The gene interaction network of DAS (*p* < 0.05) contained 198 nodes and 648 edges among 975 genes through removing unconnected nodes (Fig. 6A). And we emphasized 133 DAS genes (fold change ≥ 1.5, p value ≤ 0.05) that were imultaneously differentially expressed (fold change ≥ 1.5, p value ≤ 0.05) in the IR vs. Sham group, a total of 59 nodes and 196 edges were obtained (Fig. 6B). Using the Cytoscape software, we identified a hub gene cluster depending on the Betweenness in this PPI network. We found that genes, namely, Gsk3b, Bptf, Tpr, Gck, Fn1 and Nxf1 were located in the center of the network, and worth exploring their biological functions for further study.

## Discussion

Hepatic IR injury is a commoditized pathological scenario that occurs frequently in liver diseases and surgical procedures. Additionally, IR-induced hepatic injury can alter gene expression through its effects on the inflammatory response, oxidative stress, and apoptosis^15^. Transcriptional initiation and alternative pre-mRNA splicing are also necessary for generating the diversity of gene expression. Post-transcriptional regulation, particularly through mRNA splicing, plays a pivotal role in the pathogenesis of hepatic IR injury, contributing to the damage of various organs by modulating gene expression patterns^16,17^. However, the underlying mechanisms of AS events in hepatic IR injury and its contribution to the pathology of various organs remain not fully understood.

In this study, we used RNA-seq data to analyze the AS patterns, which differed significantly, and DAS genes in hepatic IR injury for the first time. Among 17,979 identified transcripts from RNA sequencing data, 2,171 DET were identified in the IR and sham groups, of which 1249 DAS genes were found. The results showed that the prevalence and importance of AS regulation in hepatic IR injury. Among the five major AS event types, the proportion of SE events was the highest among the annotated isoforms, accounting for 66.88%; while the A5SS events have the lowest share at 6.01%. Previous studies showed that immune evasion in hepatocellular carcinoma was facilitated by the activation of β-catenin through the CRISPR-Cas9-mediated SE event^18^. Consequently, the development and progression of hepatic IR injury may be significantly influenced by the SE event of the pre-mRNAs.

The GO terms and KEGG pathway enrichment analysis used in the following sections were performed to identify the molecular roles of gene isoforms and hub AS events. The primary roles of the DAS genes examined by GO functional annotation were in nitrogen compound metabolic process, metabolic process and organic substance metabolic process.

The KEGG pathways enrichment analysis revealed that the DAS genes were primarily linked to ATP-dependent chromatin pathway, phosphatidylinositol signal pathways, protein processing pathways and splicesome signaling pathway. The inflammatory fragment of excessive activation of complement and coagulation cascades is one of the main triggering factors of hepatic IR injury. Previous studies have reported that many differentially expressed genes in HIRI were enriched in multiple metabolic pathways^19^. The production of glycolytic ATP post-reperfusion hinders the necrotic destruction of IR-induced liver cells^20^. And the IR injury primarily modifies liver functions. within metabolic routes, encompassing the metabolism of energy, lipids and amino acids, inflammation response and oxidative stress^3^. These data have provided compelling evidence that AS events played a crucial role in the multifaceted development and progression of IR-induced hepatic injury.

Indeed, alternative RNA splicing plays a dual role in liver development and regeneration, both preserving the differentiated state of cells and regulating adaptive changes^21,22^. Many genes with specific AS events in mice exhibit differential expression between IR groups and sham groups, which may be due to the effect of IR-induced liver dysfunction. Accumulating data highlight the significance of alternatively spliced mRNA isoforms in the liver pathological changes, including inflammatory response, oxidative stress and apoptosis, which have been recently reported to be associated with AS of immune system-related transcripts, including encoding cluster of differentiation antigen-presenting molecules and interleukins^23^. Previous studies suggested that interleukin-32 with nine alternative spliced isoforms was induced mainly by pro-inflammatory cytokines to ameliorate ethanol-induced liver damage and high fat diet-induced metabolic disorder^24,19^. The expression patterns of hepatitis B virus RNA undergoing AS events were modulated during liver injuries, which would generate a viral protective effect to dampen immune-mediated inflammation and thereby reduce liver damage^25^. In addition, numerous studies have found that differential AS events contributed to oxidative stress during mouse liver injury^26,27^. Our data showed the relative abundance of hepatic antioxidant metabolic genes, such as Glutaredoxin 2 (Glrx2) with SE events and Txnrd1with SE and RI events, which played crucial roles in protecting against the oxidative damage^28^. Glrx2 maintained the mitochondrial redox homeostasis and protected hepatocytes from IR-induced liver injury and acetaminophen-induced hepatotoxicity by suppressing and inflammation, oxidative stress and apoptosis^29,30^. The antioxidative protein, thioredoxin reductase 1(Txnrd1) exhibits four transcript variants that resulted from AS events. These variants play a role in antioxidant defense mechanisms and regulate cell proliferation^31,32^. Furthermore, DRAK2 exacerbated the progression of non-alcoholic fatty liver disease progression by regulating the AS of mitochondrial function-related genes^21^. RBM15 binds RNA, thereby influencing posttranscriptional modifications such as alternative RNA splicing and protein translation, megakaryocyte differentiation and liver maturation^33,34^. Knockdown of RBM15 significantly inhibited the proliferation and invasion, and promoted cell apoptosis^35^. Thus, altered RNA splicing within the liver could potentially alter the progression of hepatic IR injury.

Furthermore, the latest research offers a compelling biological justification for the creation of innovative treatment approaches that concentrate on alternatively spliced isoforms in liver disorders. Small molecule inhibitors that modulate the activity of eukaryotic splicesomes by targeting the alternative RNA splicing of liver diseases have been developed and tested in cancer models^36,37^. The Bcl-2 family of proteins depends on alternative RNA splicing and splicing factors for their proteomic complexity and functional diversification as key regulators of mitochondrial-mediated apoptosis.

The small-molecule inhibitors influence the tumor cells’ ability to evade apoptosis by broadly binding to the Bcl-x(L) and Bcl-2 proteins^38^. The creation of splice variant-specific siRNAs or snRNAs, splice-switching anti-sense oligonucleotides, and small molecules that target splicing factors and splicing factor kinases are examples of alternative therapeutic strategies^39–41^. The search for liver IR-inducing splicing event mechanisms will aid in the creation of fresh approaches to treatment. Studying the role of AS in HIRI may help us to better understand the molecular mechanisms of liver injury and repair. Understanding the role of AS in HIRI may also help us to identify and develop biomarkers for early diagnosis and prognostic assessment. Recent studies have shown that the AS of Ceacam1 affects IR injury in mouse and human livers^42^. This suggests that by intervening in the splicing process, it may help protect the liver from IR injury. However, due to the limited sample size of this study, this conclusion needs to be further proved with a larger sample in the future.

## Conclusion

In this study, we initially characterized the landscape of AS events in IR-induced hepatic injury in mice using RNA-Seq data analysis. Utilizing rMATS, we found that SE was the most prevalent type of AS event. DETs and DAS genes are likely to play pivotal roles in hepatocyte development and maturation. This insight could facilitate a more profound understanding of the molecular events driving the pathology of IR-induced hepatic injury and potentially reveal new therapeutic targets in the foreseeable future.

## Materials and methods

### Animals and hepatic IR mouse model

Male C57BL/6 mice (8–10 weeks, weighing 20 ± 4 g) were purchased from Charles River Lab Animal Center, (Beijing, China). All mice were group housed under the specific-pathogen free (SPF) room in a temperature-controlled environment (22 ± 3 °C) with a 12-hour reversed light/dark cycle.

A total of 10 male mice were randomly divided into the following groups (five mice per group): the sham group and the IR group. The models of hepatic IR in mice were performed as described previously to induce the 70% warm ischemia followed by reperfusion. Briefly, mice were anesthetized with sodium pentobarbital (60 mg/kg). A midline laparotomy was performed, and an atraumatic clip was used to interrupt the blood supply of the liver left lateral and median lobes at their bases. The IR group was performed for 90 min of partial hepatic ischemia and 6 h of reperfusion. The sham group underwent the same surgical procedures without vascular clamping. After the indicated period of reperfusion, all mice were humanely euthanized by cervical dislocation without any chemical agent, blood samples and liver tissues were harvested for further analysis^20^.

### DET and DAS analysis

According to the criteria of (log2|fold change| ≥ 0.585/log2|fold change| ≤ -0.585 and *p* ≤ 0.05), the DETs were identified based on the exon per million mapped reads (FPKM) values (FPKM ≥ 0.5). DAS genes in IR group and sham group were screened out using rMATS. The DAS genes were defined as a threshold of |lncLevel difference| >0.05 and the false discovery rate (FDR) < 0.05. The p-value was calculated by likelihood-ratio test that the difference between two sample groups exceeded a given threshold: default 1% difference.

### AS analysis

The AS events and their corresponding to all five major types of AS patterns (A3SS, A5SS, MXE, RI, and SE) from RNA-seq data with paired replicates were detected and calculated using the rMATS multivariate analysis tool. At the threshold of |Δψ|>5% and FDR of ≤ 0.05 for between-group difference, the read counts mapped to the splice junction and to the exon body were used as the reference annotation for rMATS.

### Total RNA isolation and RNA-seq library construction

Total RNA extracted from liver samples using Trizol reagent (Ambion) was qualified and quantified with an agarose gel electrophoresis and a Nanodrop ND-1000 spectrophotometer^20^. By using the oligo (dT) magnetic beads, the intact mRNA was enriched (NEB Next Poly(A) mRNA Magnetic Isolation Module) and rRNA was removed (RiboZero Magnetic Gold kit). The purified RNA (2ug total RNA) was used to construct the RNA-seq library using KAPA Stranded RNA-Seq Library Prep Kit (Illumina) as the following steps: the first strand cDNA synthesis was carried out by using a partial adaptor sequence with random primer; then the dUTP was incorporated into the second cDNA strand to render the RNA-seq library strand-specific. The constructed RNA-seq libraries were qualified with Agilent 2100 Bioanalyzer and precisely quantified by qRT-PCR assays. The libraries composed of different samples were sequenced with an Illumina NovaSeq 6000 system platform. Benjamini-Hochberg procedure was designed to account for multiple comparisons (FDR < 0.05).

### Quantitative real-time polymerase chain reaction (qRT-PCR)

Total RNA of liver tissue from IR group and sham group was extracted using an RNA Miniprep Kit (Axygen) and quantified through spectrophotometry (Nanodrop ND-1000 spectrophotometer)^1^. According to the manufacturer’s instructions, RNA was reverse-transcribed into complementary DNA by using the ReverTra Ace qPCR RT reagent Kit. Quantitative RT-PCR was performed using the Roche FastStart Universal SYBR Green Master Mix (ROX). The qRT‒PCR procedure was performed as follows: holding initially at 94 °C for 4 min, followed by 40 cycles of 94 °C for 15 s (denaturation) and 58 °C for 45 s (annealing and extension). All samples were normalized to the housekeeping gene β-actin, and the targeted gene expression for relative quantification was calculated by the 2^−ΔΔ Ct^ method. Each PCR was repeated three times with three biological replicates. All primers of target genes used in this study are listed in Table S3.

### Gene interaction network construction

In order to further explore the functional interactions and associations of the AS genes in hepatic IR injury, the corresponding gene identifier names of AS events the protein/gene interaction network of the corresponding gene identifier names of AS events was constructed through STRING (Search Tool for the Retrieval of Interacting Genes/Proteins) in our study. STRING database (https://cn.string-db.org/) could predict protein interactions and summarize the complex interactions in a network view. The minimum required interactions with a combined score > 0.4 were chosen to construct PPI networks for the DAS genes. Network analysis was performed using Cytoscape v3.8.2 to identify the hub genes.

### Ontological and pathway analysis

Annotation of the three functionality domains: biological process (BP), cellular component (CC), and molecular function (MF) were determined using the significance analysis of GO enrichment. The gene clusters and biological functions were determined using the KEGG pathway analysis, which was ranked by the enrichment scores. GO and KEGG functional enrichment analysis of DAS genes were performed using Fisher’s exact test and the clusterProfiler package of R software to present the categorical data.

### Statistical analysis

The image processing and base recognition were performed by using Solexa pipeline v1.8 (Off-Line Base Caller software, v1.8). Quality control of raw RNA-seq reads was examined with FastQC software according to Q30 standards. Sequence reads were mapped onto the reference genome sequences using HISAT2. The fragments per kilobase of Fragments Per Kilobase of gene/transcript model per Million mapped fragments (FPKM) values of transcription levels and gene expression levels were calculated using the estimate R package Ballgown. All data in this study were presented as mean ± SD. A p-value of less than 0.05 was considered statistically significant for all the analyses.

## Electronic supplementary material

Below is the link to the electronic supplementary material.

Supplementary Material 1

Supplementary Material 2

Supplementary Material 3

## Data Availability

The data used to support the findings of this study are available from the corresponding author upon request. RNA-seq data that support the findings of this study have been deposited in the Gene Expression Omnibus (GEO) database (https://www.ncbi.nlm.nih.gov/geo/), accession codes GSE190216.
