# Supplementary material for: Genome-wide analysis of alternative splicing differences in hepatic ischemia reperfusion injury
Source: Sci Rep. 2024 Dec 28;14:31349. doi: 10.1038/s41598-024-82846-1 (PMC11682299; doi:10.1038/s41598-024-82846-1)
Supplement: Supplementary file 1 — Supplementary Material 1 [file 41598_2024_82846_MOESM1_ESM.docx]

**Table S1**. Summary of RNA-seq results of reads.

| Samples | Reads Count | Bases Num | Bases Num (Q≥30) | Q30(%) |
| --- | --- | --- | --- | --- |
| IR-1 | 39526074 | 5928911100 | 5461702248 | 92.12% |
| IR-2 | 36802584 | 5520387600 | 5065365280 | 91.76% |
| IR-3 | 41578866 | 6236829900 | 5716372325 | 91.66% |
| IR-4 | 42016504 | 6302475600 | 5788097418 | 91.84% |
| IR-5 | 43628212 | 6544231800 | 6061465021 | 92.62% |
| Sham-1 | 38856626 | 5828493900 | 5320480537 | 91.28% |
| Sham-2 | 46899416 | 7034912400 | 6475996711 | 92.06% |
| Sham-3 | 37601238 | 5640185700 | 5203332182 | 92.25% |
| Sham-4 | 36341984 | 5451297600 | 5025634205 | 92.19% |
| Sham-5 | 49752054 | 7462808100 | 6879684682 | 92.19% |

**Table S2.** The numbers of differential AS events.

| AS_Event | A5SS | A3SS | SE | RI | MXE |
| --- | --- | --- | --- | --- | --- |
| Up | 375 | 439 | 2694 | 986 | 403 |
| Down | 222 | 289 | 2352 | 233 | 605 |
| P_value | 597 | 728 | 5051 | 1221 | 1010 |
| Total | 4248 | 6687 | 58975 | 4919 | 8219 |

**Table S3.** Primer sequences for real-time quantitative PCR.

| Gene | Forward sequence | Reverse sequence |
| --- | --- | --- |
| GAPDH | TGGATTTGGACGCATTGGTC | TTTGCACTGGTACGTGTTGAT |
| Gabpb2 | GAAATTCTCTTGACTCCTCGACC | AGGCACAGTTAGAACTTGCTG |
| Smg1 | ACAGAGTAATGAGATGCGTGACG | CAAGCTGCCGAGCAAAACAC |
| Tnrc6c  Mettl17  Smpd4  Kcnt2  D16Ertd472e  Rab3gap2  Echdc2  Ssx2ip | CATCCCAGCCGAAGTTCTTCT  GCCAGGACACCGAGATTTCTG  CACACTAGCCTCTTGAAGCGA  AAAGATTTTGCCCCAAATTGTCC  TTGCAGTGTTTGTAAACTGGGA  AGGCTCAGCATTCCAAGGAC  CGCAATGCCTTGGGGAATG  ATGGGAGATTGGATGACTGTGA | CACCCTGTTGATCCTTTCCCA  AATCCGACCCGTTGTCTACCT  TGTAGAACCTCCAACTTGGCAT  CTCCCGTAAGTCTTCTGCCAC  AGCAGTCTTTATGGCCTCTCA  GGTCTTTCCAATGAGTGTTTGC  TTCACCGCACTTCTGAAGAGC  TGGGAGTACAAACTGGACGGA |
