# Supplementary figures and images for: Genome-wide analysis of alternative splicing differences in hepatic ischemia reperfusion injury
Source: Sci Rep. 2024 Dec 28;14:31349. doi: 10.1038/s41598-024-82846-1 (PMC11682299; doi:10.1038/s41598-024-82846-1)

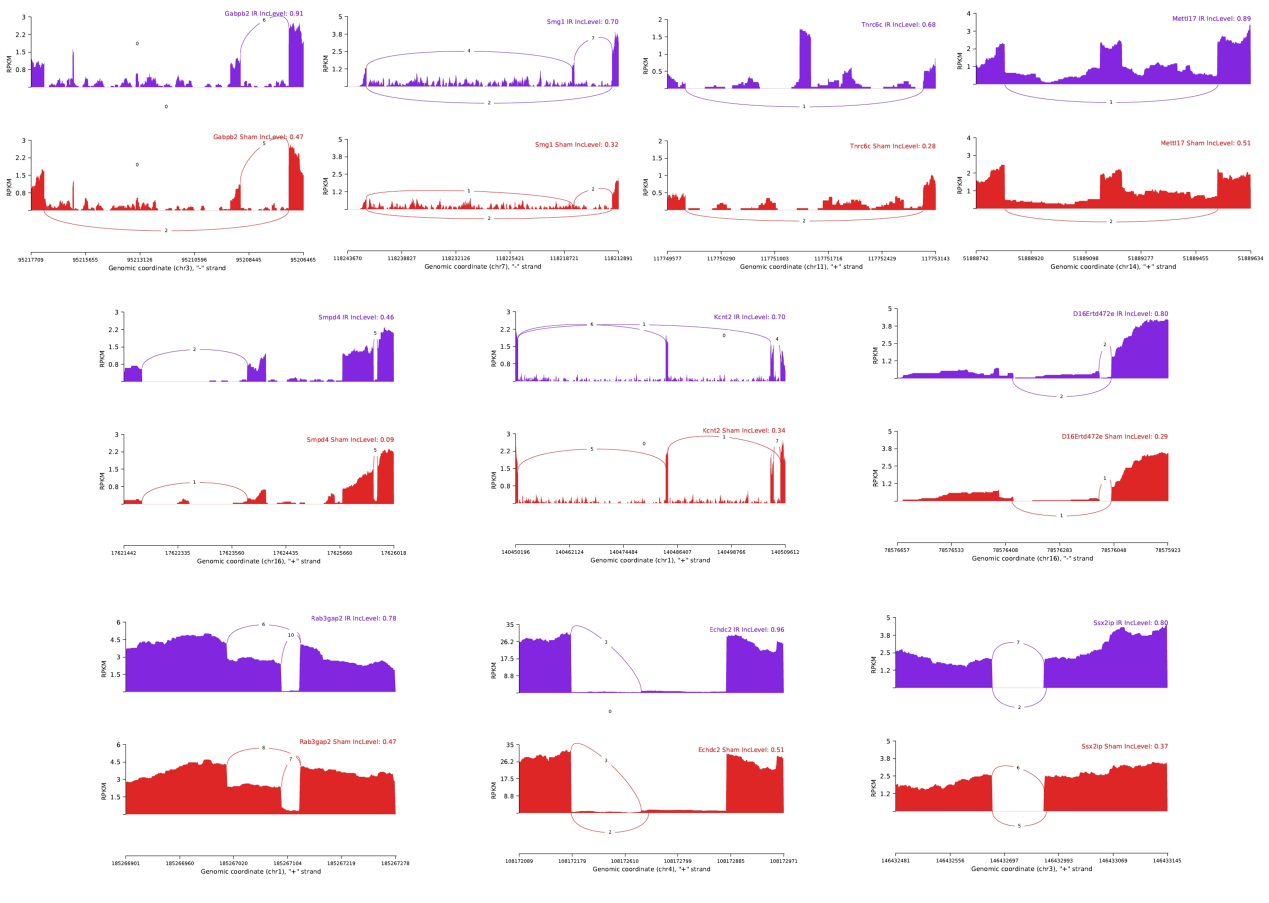


**Supplemental Fig. 1** RNA sequencing read density plot.

Supplement: Supplementary file 2 — Supplementary Material 2 [file 41598_2024_82846_MOESM2_ESM.docx]

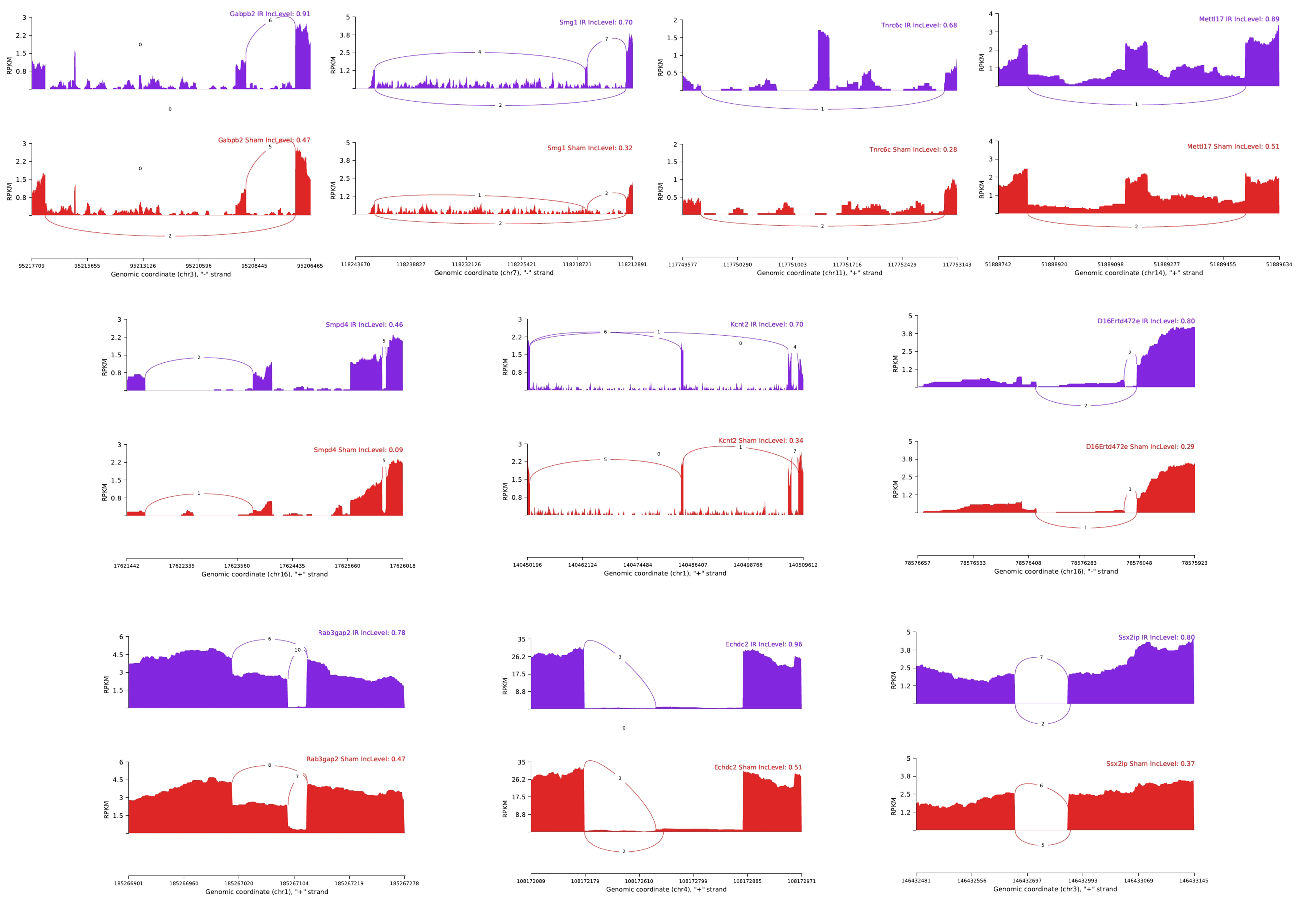

Supplement: Supplementary file 3 — Supplementary Material 3 [file 41598_2024_82846_MOESM3_ESM.png]
